# Determination of cardiac output from pulse pressure contour during intra-aortic balloon pumping in patients with low ejection fraction

**DOI:** 10.1007/s10877-019-00320-0

**Published:** 2019-05-14

**Authors:** Jos R. C. Jansen, Marcelo B Bastos, Pat Hanlon, Nicolas M. Van Mieghem, Ottavio Alfieri, Jan J. Schreuder

**Affiliations:** 1 Department of Intensive Care Medicine, Leiden University Medical Center, P.O. Box 9600, 2300 RC Leiden, The Netherlands; 2 Department of Interventional Cardiology, Thoraxcenter, Erasmus Medical Center, Rotterdam, The Netherlands; 3 Teleflex Medical/Arrow, Interventional, 16 Elizabeth Dr, Chelmsford, MA 01824 USA; 4 Department of Cardiac Surgery, San Raffaele University Hospital, Via Olgettina 60, 20132 Milan, Italy

**Keywords:** Cardiac output, Stroke volume, IABP, Pulse contour, Conductance

## Abstract

Evaluation of a new Windkessel model based pulse contour method (WKflow) to calculate stroke volume in patients undergoing intra-aortic balloon pumping (IABP). Preload changes were induced by vena cava occlusions (VCO) in twelve patients undergoing cardiac surgery to vary stroke volume (SV), which was measured by left ventricular conductance volume method (SVlv) and WKflow (SVwf). Twelve VCO series were carried out during IABP assist at a 1:2 ratio and seven VCO series were performed with IABP switched off. Additionally, SVwf was evaluated during nine episodes of severe arrhythmia. VCO’s produced marked changes in SV over 10–20 beats. 198 paired data sets of SVlv and SVwf were obtained. Bland–Altman analysis for the difference between SVlv and SVwf during IABP in 1:2 mode showed a bias (accuracy) of 1.04 ± 3.99 ml, precision 10.9% and limits of agreement (LOA) of − 6.94 to 9.02 ml. Without IABP bias was 0.48 ± 4.36 ml, precision 11.6% and LOA of − 8.24 to 9.20 ml. After one thermodilution calibration of SVwf per patient, during IABP the accuracy improved to 0.14 ± 3.07 ml, precision to 8.3% and LOA to − 6.00 to + 6.28 ml. Without IABP the accuracy improved to 0.01 ± 2.71 ml, precision to 7.5% and LOA to − 5.41 to + 5.43 ml. Changes in SVlv and SVwf were directionally concordant in response to VCO’s and during severe arrhythmia. (R^2^ = 0.868). The SVwf and SVlv methods are interchangeable with respect to measuring absolute stroke volume as well as tracking changes in stroke volume. The precision of the non-calibrated WKflow method is about 10% which improved to 7.5% after one calibration per patient.

## Introduction

Intra-aortic balloon counter pulsation (IABP) to support cardiac function has been well established during the last four decades [1–7]. Main effects of properly timed IABP are afterload reduction and diastolic aortic pressure augmentation increasing coronary flow [4, 5, 8, 9]. Left ventricular (LV) volume and LV end-diastolic pressure may decrease during IABP, whereas cardiac output, ejection fraction (EF) and coronary flow may increase [3–7]. Pantalos and colleagues [10] showed that measured diastolic augmentation and afterload reduction varied with pressure source for IABP. Timing related to pressure measured in the aorta with a high-fidelity pressure sensing device resulted in a reduction in afterload [8, 9].

Monitoring cardiac function by cardiac output (CO) measurement is an obvious choice [11]. In critically ill patients cardiac output may vary acutely and continuously due to arrhythmia or therapeutic interventions. In these cases, beat-to-beat monitoring of CO may give acutely essential information about responses to treatment. Real time CO measurements in mechanically assisted patients are particularly important and may allow early determination of the effectiveness of IABP or the need for escalation to another type of mechanical cardiac support.

Many bedside devices which monitor stroke volume (SV) and cardiac output require additional catheters and clinician time to obtain these data and lack beat-to-beat values. In contrast, pressure pulse contour analysis allows for instantaneous beat-to-beat SV and CO derived from the pressure source used to control the IABP. In its ultimate form the method could be incorporated into the IABP device. Multiple attempts have been made to derive SV and CO from the arterial pressure contour [11–13]. The major shortcomings were related to suboptimal models of the arterial system, changes in arterial compliance, pressure distortions of the aortic and radial artery pressure caused by IABP and arrhythmia.

The aim of our study is to describe a solution for these problems and to evaluate the new WKflow pulse contour cardiac output method in patients with a low ejection fraction undergoing cardiac surgery and requiring prophylactic IABP support. Temporary vena cava occlusion procedures were performed to change SV over a marked range. The agreement between beat-to-beat stroke volume values measured by the left ventricular conductance volume method and WKflow method was studied in stable conditions and during significant arrhythmia.

## Patients and methods

The study was approved by the ethics committee of the San Raffaele University Hospital, and written informed patient consent was obtained from all patients. The current study partially used hemodynamic data from the same patients reported in another study but analyzed different protocol-based measures [9]. Patients with aortic aneurysm, severe peripheral vascular diseases, and aortic or mitral valve insufficiencies were excluded.

The effects of vena cava occlusions (VCO) were studied in twelve patients (NYHA class II to IV), 57 to 74 years old, with EF 21 to 39% undergoing CABG and or LV aneurysmectomy. All patients received opioid based anesthesia. Five patients received steady-state infusion of dobutamine (dosage < 5 μg kg^−1^ min^−1^). All subjects were mechanically ventilated with volume-controlled ventilation adjusted to achieve normocapnia, with tidal volumes of 7–12 ml kg^−1^, frequencies of 12–14 min^−1^ and 5 cm H_2_O positive end-expiratory pressure.

### Instrumentation

The IAB catheters (Narrowflex 8F LightWAVE; Arrow Intl, Reading, PA) equipped with a fiber-optic high-fidelity pressure transducer at the tip were positioned under transesophageal echo control in the descending thoracic aorta at 2 cm distal to the left subclavian artery. The IAB catheters were connected to an IABP device (AutoCat 2 Wave, Arrow Intl). Standard procedures were followed to time inflation and deflation of the IAB. Thermodilution catheters (Edwards Lifesciences, Irvine, CA) were inserted into the pulmonary artery. Conductance volume catheters equipped with a high-fidelity micromanometer (F7; CD Leycom, Hengelo, The Netherlands) were positioned into the LV along the long axis via a pulmonary vein. The conductance pressure–volume catheters were connected to a cardiac function analyzer (CD Leycom) to measure ventricular volumes [8, 9, 14] using dedicated software (Conduct NT v3.18, CD Leycom). The conductance volume method is based on measuring time-varying electrical conductance of 5 to 7 ventricular blood segments, delineated by selected catheter electrodes. Correct positioning of the pressure–volume catheter was verified by transesophageal echocardiography and by inspection of the segmental volume signals. Parallel conductance was assessed by injection of 10 ml hypertonic saline (6%) into the pulmonary artery [8, 9, 14]. Absolute LV volumes were calculated by matching effective conductance SV with simultaneously measured thermodilution SV and by subtracting parallel conductance from total conductance volume.

### Stroke volume by aortic pressure curve analysis

**Fig. 1:**
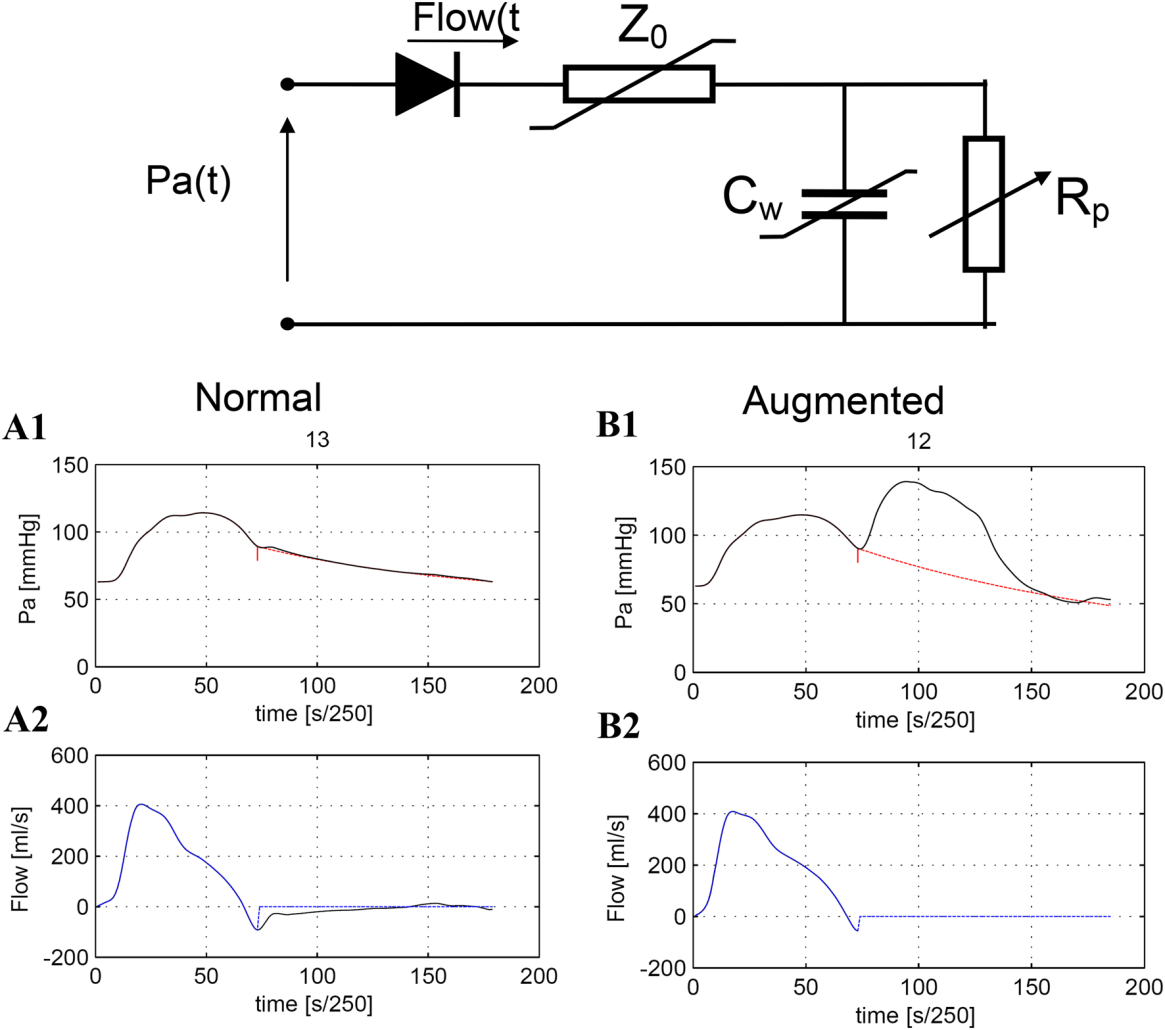
Aortic flow simulated from aortic pressure with WKflow during a normal (**A**) and an augmented (**B**) heartbeat. In panel **A1** a recording of a single beat of aortic pressure measured with a high fidelity fiber optic pressure transducer on the IAB catheter, black line. In panel **A2** the resulting flow is plotted. With this simulated flow an aortic pressure beat is simulated, backwards, see **A1** dotted red line which falls together with the measured pressure line. In an optimization, peripheral resistance (Rp) of WKflow is adjusted to create an optimal fit between measure and simulated pressure at end of diastole. The vertical narrow line indicates the moment of aortic valve closure which was derived from the first local minimum in aortic flow. In panels **B1** and **B2** the same simulation is shown during IABP. Here the red dotted line represents the simulated aortic pressure curve without augmentation. From this curve systolic, diastolic and mean arterial blood pressure can be calculated

The WKflow method uses a nonlinear, time varying three element Windkessel model, similar to the Modelflow method [15]. The model includes aortic characteristic impedance (Z0), arterial compliance (Cw), and systemic vascular resistance (Rp) (Fig. 1). Time varying Z0 and Cw depend on the aortic cross-sectional area, which can be estimated from time varying arterial pressure, age, and gender by means of the arctangent model of Langewouters et al. [16]. To obtain the correct value of Rp for the beat under study a number of steps occur. First, model aortic blood flow is simulated with Rp from the former beat. Simulated blood flow during diastole is set to zero (assuming no aortic valvular leakage in this phase). A time varying aortic pressure is calculated with the simulated flow and the Windkessel parameters Rp, Cw and Z0. Next to the beat under study, Rp is adjusted by an optimization procedure in which the difference between simulated and measure aortic pressure at the end of the beat is minimized. So the optimization does not include aortic pressure during augmentation. With this scheme, the model can follow changes in systemic peripheral resistances that occur with a time constant which is typically about 10 s. After optimization the dicrotic notch is detected at the first local minimum in the aortic flow signal after peak flow [8, 9, 17, 18] (Fig. 1). Integrating the flow from pressure upstroke to dicrotic notch gives stroke volume (area under flow curve). Cardiac output is found by multiplying stroke volume by heart rate. Furthermore, the simulated aortic pressure can be used to calculate mean, diastolic and systolic aortic pressure.

### Stroke volume during left ventricular volume changes

In the absence of mitral regurgitation the amount of blood ejected by the left ventricle, i.e. stroke volume is equal to the change in LV volume during systole. With the WKflow method we accurately define begin of systole by the aortic pressure upstroke and the end of systole by detecting aortic valve closure from the simulated aortic flow pattern [8, 9, 17, 18].

### Measurement protocol

Measurements were performed during vena cava occlusions before or after cardio-pulmonary bypass during IABP at a 1:2 assist ratio and in a subgroup of patients with IABP not pumping. ECG, LV pressure (Plv), aortic pressure (Pao), intra-aortic balloon pressure (Piabp) and LV volume (Vlv) signals were measured and digitally sampled at a rate of 250 Hz and stored on computer disk. Data recorded during marked arrhythmia, was used to evaluate the robustness of the WKflow model.

### Calculations and statistics

After confirming a normal distribution of data with the Kolmogorov–Smirnov test, agreement between SVlv, SVwf, as well as agreement in changes in stroke volume was evaluated with Bland–Altman statistics. The agreement between SVlv and SVwf was computed as the bias (i.e. low bias is high accuracy) and standard deviation (SD) (i.e. low SD high precision), with the limits of agreement (LOA) computed as the bias ± 2SD. Percentage precision or coefficient of variation (COV) was computed as [COV = 100 * (SD/mean)]. Low COV represents high precision. In addition linear regression between SVlv and SVwf is performed. The slope and goodness of fit by assessing R^2^ are calculated. After confirming equality of variance by Levene’s statistical test of the results of VCO with the IABP in 1:2 mode and the results of VCO without IABP were merged.

The ability to monitor changes in stroke volume (ΔSV) due to our interventions is calculated by subtracting the beat-to-beat stroke volume values from the beat SV values at start of the interventions. These changes are expressed in percentages. When the change in values of SVwf was in the same direction as those found for SVlv we regarded this change a ‘positive trend’, whereas, a ‘negative trend’ was one where these changed in opposite directions. Ideally, only positive scores should be present. These scores were analyzed using 2 × 2 tables and presented as percentages. Separate scores for clinically relevant changes of SVlv values (5 to 10%) were counted. A *p* value < 0.05 was considered statistically significant. Unless otherwise stated, data are presented as mean ± SD.

## Results

**Fig. 2:**
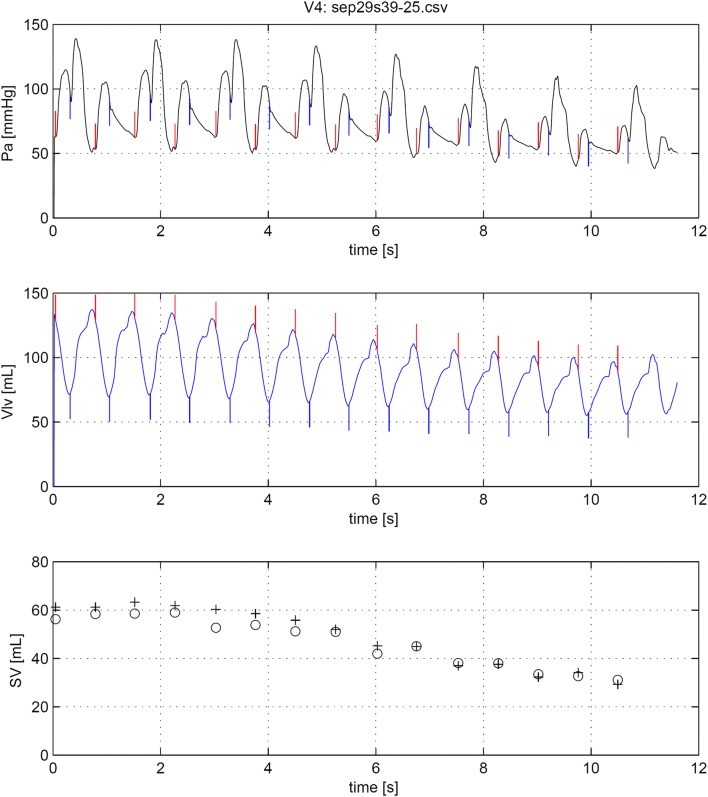
Example of the effect of a vena cava occlusion during IABP in a 1:2 mode. In upper panel the aortic pressure (Pa) is plotted. The narrow vertical upwards plotted lines indicated start of ejection (begin systole) and the downwards lines the moment of aortic valve closure (end systole). In the middle panel the left ventricular volume (Vlv) signal is plotted. The volume variation during systole is equal to stoke volume. Begin and end systole are indicated. In the lower panel beat-to-beat stroke volume (SV) derived from Vlv (open circles) and from WKflow (plus sign) are plotted at begin systole of each beat

Demographics and main hemodynamic data of the patient population are given in Table 1. Twelve series of measurement with VCO procedures were carried out during IABP 1:2 assist ratio. Two series were not suitable for analysis due to noise in the aortic pressure signal caused by IAB catheter whip. In addition, in six patients, seven VCO procedures were performed without IABP support. Vena cava occlusions may produce large changes in SV by reducing venous return to the heart. This resulted in marked changes in left ventricular stroke volume over 20 to 30 beats, see Fig. 2. In total, hundred ninety-eight paired data sets of SVlv and SVwf were obtained. This data were normally distributed.

**Table 1:** Demographic data all patients

No	Gender	Age	Height	Weight	HR	EFc	SVes	Pao,sys	Pao,dias	Remarks
		Years	cm	kg	min^−1^	%	ml	mmHg	mmHg	
1	m	63	165	63	82	31	41	80	48	CABG and LV aneurysmectomy
2	f	58	161	73	102	31	57	87	61	CABG recordings after mitral valve repair
3	f	66	165	73	107	28	53	94	58	CABG and LV aneurysmectomy
4	m	62	175	76	97	32	61	97	59	CABG and BiV pacing
5	m	73	175	75	82	32	69	129	65	LV aneurysmectomy
6	m	67	168	65	78	25	40	95	63	CABG: small MV regurgitation observable
7	m	70	160	66	85	39	61	123	55	CABG and LV aneurysmectomy
8	m	64	165	65	46	32	69	89	49	LV aneurysmectomy
9	m	57	175	80	94	21	59	116	65	CABG and dilated cardiomyopathy
10	m	62	178	77	97	23	43	118	73	LV aneurysmectomy
11	m	74	174	82	67	33	61	85	55	LV aneurysmectomy
12	m	64	170	69	83	25	48	99	42	CABG and dilated cardiomyopathy
Mean		65.0	169.3	72.0	85.0	29.3	55.2	101.0	57.7	
SD		5.3	6.1	6.3	16.7	5.1	10.1	16.3	8.6	

All variables recorded at start of the observation period

*HR* Heart rate, *EFc* ejection fraction, *SVes* stroke volume, *Pao,sys* systolic aortic pressure, *Pao,dias* diastolic aortic pressure, *CABG* coronary artery bypass grafting, *MVR* mitral valve repair, *BiV* bi-ventriclular pacing, *LV* left ventricular

### Regression analysis

**Fig. 3:**
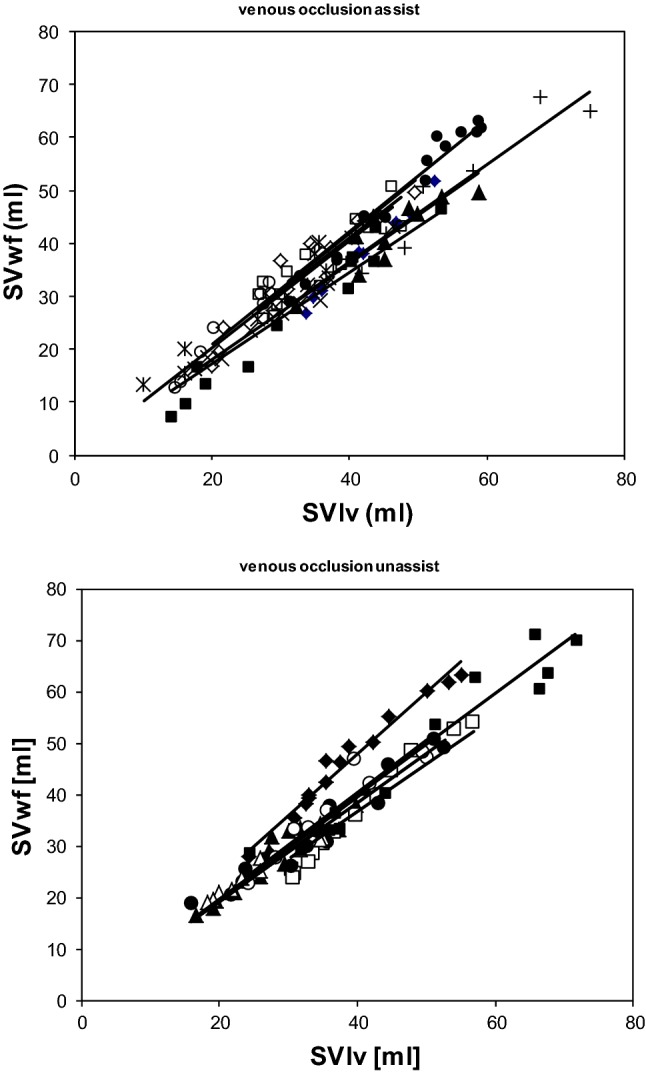
Regression between left ventricular stroke volume (SV) measured by the conductance method (SVlv) and WKflow method (SVwf) during vena cava occlusion during during IABP in 1:2 mode (upper panel) and with IABP switched off (lower panel). Each line is the result of a linear fit through the beat-to-beat SVlv and SVwf data points obtained during each single vena cava occlusion procedure

In each individual patient there is a strong relation between SVlv and un-calibrated SVwf (Fig. 3). During IABP in 1:2 assist the slope of the augmented beats, 0.994 ± 0.107, is not significantly different from the following assisted beats 0.994 ± 0.053 (p = 0.852). Combining all beats during IABP the slope is 0.997 ± 0.088, correlation coefficient R^2^ is 0.891 ± 0.070 and not different (p = 0.912) from unity. For the observation made without IABP the averaged slope is 0.978 **± **0.128 and R^2^ is 0.899 ± 0.108. This slope is not different from unity, p = 0.661.

### Agreement between SVconductance and WKflow method

The results of Bland–Altman analysis comparing SVlv and non-calibrated SVwf during vena cava occlusion with IABP in 1:2 mode are shown in Table 2. Bias between SVlv and SVwf is 1.04 ± 3.99 ml (small but significantly different from zero, p = 0.007). The limits of agreement (LOA) range from − 6.94 to 9.02 ml. The COV or precision is 10.9%. The results of Bland–Altman analysis during vena cava occlusion without IABP indicate an accuracy of 0.48 ± 4.36 ml (not significantly different from zero, p = 0.667) and precision is 11.6%. The LOA ranged from − 8.24 to 9.20 ml.

**Table 2:** Bland–Altman analysis of vena cava occlusions

	n	n pairs	Mean	Difference between methods			Limits of agreement		Calculated precision SVwf	
				Bias	SD		Lower	Upper	SVlv = 5%	SVlv = 10%
			ml	ml	ml	%	ml	ml	%	%
IABP 1:2	10	112	35.57	1.04	3.99	10.9	− 6.94	9.02	9.7	4.3
IABP 1:2,cal	10	112	37.02	0.14	3.07	8.3	− 6.00	6.28	6.6	0.0
No IABP	7	87	35.95	0.48	4.36	12.1	− 8.24	9.20	11.0	6.8
No IABP,cal	7	87	35.92	0.01	2.71	7.5	− 5.41	5.43	5.6	0.0
All	17	199	36.32	0.40	4.21	11.6	− 8.02	8.82	10.5	5.9
All, cal	17	199	36.45	0.13	2.83	7.8	− 5.53	5.79	6.0	0.0

IABP in 1:2 mode; cal results after calibration of WK flow stroke volume (SVwf); no IABP, IABP switched off; n, number of vena cava occlusions; bias, mean difference between stroke volume by conductance (SVlv) and SVwf. Calculated precision of SVwf assuming a precision of SVlv of, for instance, 10% results, in √(10.9^2^ − 10^2^) = 4.3%

**Fig. 4:**
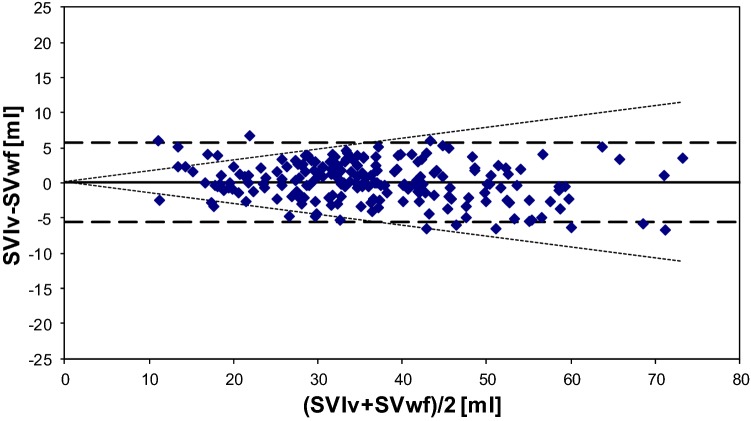
Bland-Altman plot of stroke volume (SV) by conductance method (SVlv) and once-calibrated WKflow (SVwf). Data is obtained during vena cava occlusion procedures to diminish SV during IABP in 1:2 mode as well as with IABP switched off. Limits of agreement in SV in ml (dashed line) and percentage (dotted line) are indicated. SV ranged from 10 to 75 ml

Between the group with IABP in 1:2 mode and the group without IABP we could not detect a difference in homogeneity of variance (Levine statistics p = 0.647) and in mean difference SVlv–SVwf, allowing to group all results together. The results of all VCO show a good overall agreement between SVlv and SVwf (Fig. 4 and Table 2).

### Agreement between SVconductance and WKflow method after one calibration

We performed one calibration per patient of SVwf by thermodilution. During IABP support bias or accuracy improved to 0.14 ± 3.07 ml, COV or precision to 8.3%, and LOA to − 6.00 to 6.28 ml. After calibration bias is not different from zero now (p = 0.630).

Without IABP support, calibration by thermodilution results in a markedly improvement of the agreement between SVlv and SVwf (bias 0.01 ± 2.71 ml, precision 7.5% and LOA − 5.41 to 5.43 ml). Bias is not different from zero (p = 0.331). Between the group with IABP in 1:2 mode and the group without IABP we could not detect a difference in homogeneity of variance (Levine statistics p = 0.242) and in mean difference SVlv–SVwf, allowing to group all results together. The results of Bland–Altman analysis for all observation grouped is given in Fig. 4. With no significant bias (p = 0.242) precision is 7.8% and percentage limits of agreement for the difference is 2  × 7.8 = 15.6%.

### Monitoring changes in SV during VCO

**Fig. 5:**
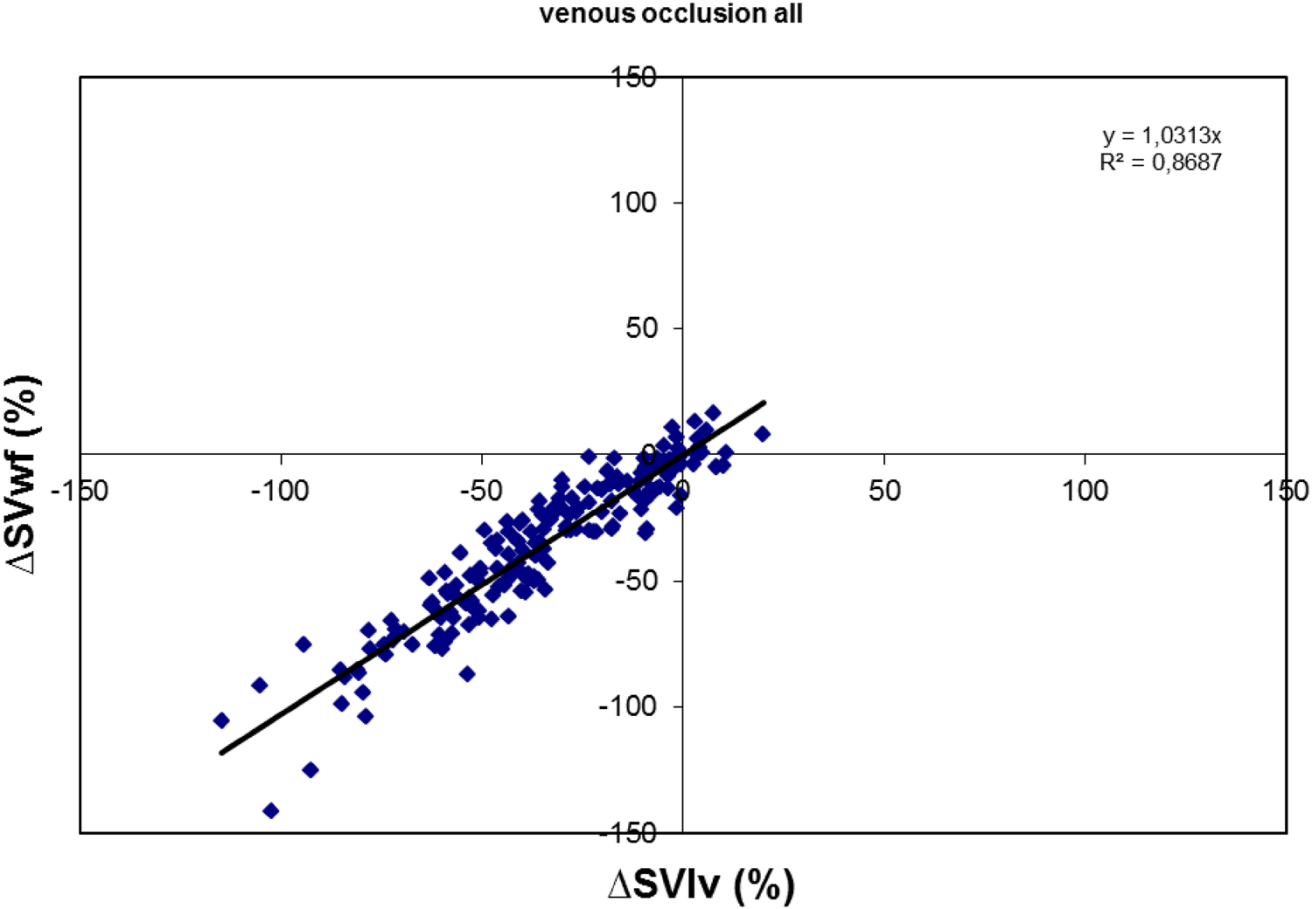
Change in stroke volume (SV) by conductance method (∆SVlv) and WKflow method (∆SVwf) obtained during vena cava occlusion. For explanation see text

The results of all beat-to-beat changes in SVlv and changes in SVwf normalized to the start of vena cave occlusion are given in Fig. 5. We found no difference in the change of SVlv and change of SVwf (p = 0.820). Regression analysis of the data for all VCO’s showed a slope of 1.031 and a goodness of fit R^2^ = 0.869.

In Fig. 5 the data points in the lower left and the upper right quadrants represent equal direction of change (correct). The data points in the upper right and lower right quadrants indicate opposite changes (incorrect). Most data points are positioned in the left under quadrant indicating that vena cava occlusion diminishes both SVlv and SVwf. Counting the number of data points with an equal direction of change in percentage of the total number of data point delivers the level of concordance between the methods. For the data presented in Fig. 5 we found concordance in 96% of the cases. This score improves if clinically irrelevant changes in absolute values < 5% are excluded from counting to 99%.

### Monitoring changes in SV during arrhythmia

**Fig. 6:**
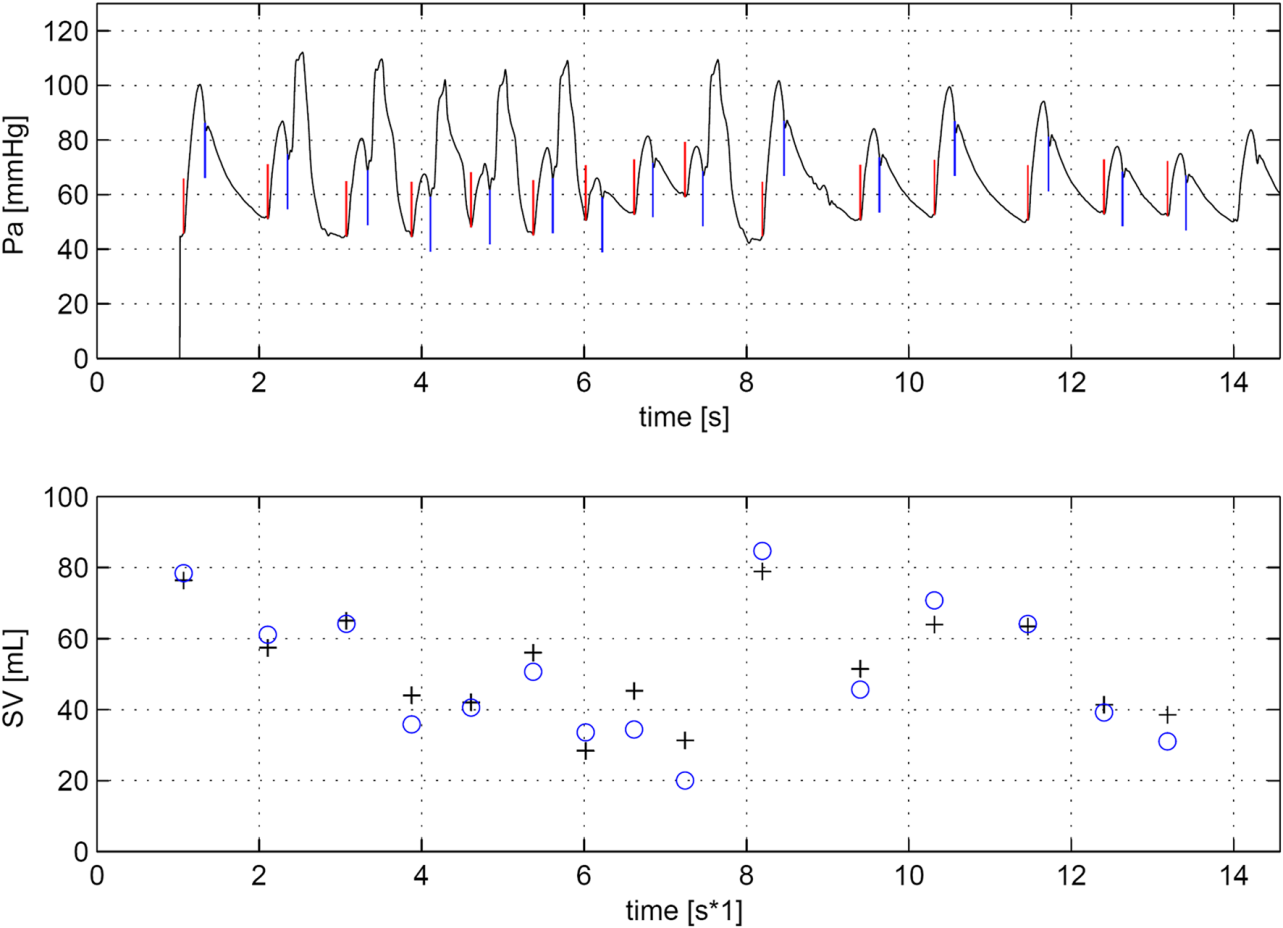
Example of irregular heartbeats. In upper panel the aortic pressure (Pa) is plotted. The narrow vertical upwards plotted lines indicate the start of ejection (begin systole) and the downwards lines the moment of aortic valve closure (end systole). In the lower panel beat-to-beat stroke volume (SV) derived from conductance method (open circles) and from WKflow (plus sign) are plotted at begin systole of each beat. A remarkable concordance between SVlv and SVwf is observable with beat-to-beat variations up to 65 ml and heart rate variating between 52 and 108 bpm

**Fig. 7:**
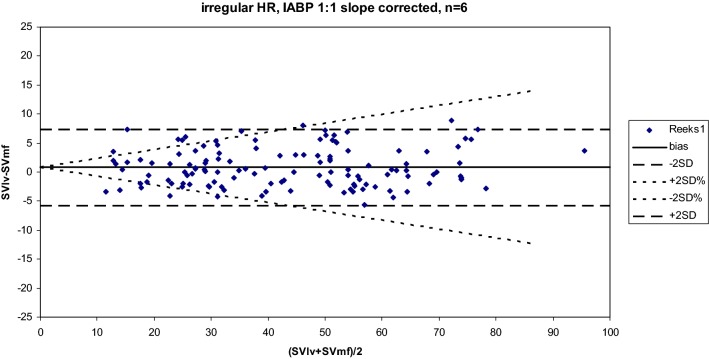
Bland-Altman analysis for agreement between the two methods after one calibration by thermodilution per patient showed a bias or accuracy of 0.828 ± 3.287 ml, precision 7.6% and LOA from − 5.75 to 7.40 ml. SV ranges from 10 up to 95 ml

During irregular heart beats and IABP assist at a 1:1 ratio we observed marked beat-to-beat changes in SVlv and SVwf varying from 20 to 85 ml, Fig. 6. Nine recordings from six different patients were analyzed with the two methods. Bland–Altman analysis for agreement between the two methods showed an accuracy or bias of 0.828 ± 3.287 ml, precision 7.6% and LOA from − 5.75 to 7.40 ml, see Fig. 7.

## Discussion

We presented a new pulse contour WKflow method for computation of beat-to-beat stroke volume from the aortic pressure signal measure with a fiber optic pressure transducer mounted on the tip of an IAB catheter. Our study reported positive results of pulse contour stroke volume during IABP under extremely challenging conditions. Good agreement was found between the paired results of beat-to-beat stroke volume values from the calibrated conductance method and the new non-calibrated WKflow method obtained during vena cava occlusion without and with IABP. Also during irregular heartbeats stroke volume by the WKflow method followed the conductance method accurately. The two methods are interchangeable with respect to measuring absolute SV as well as in monitoring changes in SV. A prerequisite for optimal WKflow estimation is optimal (correct) IAB triggering and timing during regular heart rate and arrhythmia which was provided by the AutoCat 2 Wave [8, 9].

To our knowledge this is the first study evaluating cardiac output from pulse pressure flow during intra-aortic balloon pumping using a high fidelity aortic pressure sensor in patients. However, the use of radial artery pressure in patients on IABP to estimate CO has been evaluated before [11–13]. Hoie [11] and Herlinger [12] basically described that the radial pressure excursion during IABP can be separated into the excursion of the pulse pressure (PP) due to the ejection of blood in the aorta and the excursion due to augmentation inflation of the IAB (BP). Assuming arterial compliance to be the same during both phases and knowing the volume change (BV) in the IAB these authors calculated SV = BV∙PP/BP. In this method it is assumed that the arterial system behaves as a closed system, i.e. during the ejection of blood and during IAB augmentation no blood is leaving the arterial system, which in reality occurs. To minimize for this drainage deflation pressure was chosen to measure BP because at the end of the beat the drainage of blood is lowest. To our knowledge the method is not in use currently. Lorsomradee and colleagues [13] used a standard commercial available pulse contour system (Vigileo monitor, Edwards) connected to the radial artery pressure line to monitor CO. Based on their results the authors concluded that application of standard pulse contour methods for estimation of CO during IABP may become less reliable. In contrast: The behavior of WKflow method is primarily related to aortic pressure and model parameters during systole, a phase in the heart cycle where aortic pressure is not directly disturbed by IABP augmentation.

### Choice of reference method

Thermodilution is the reference CO (COtd) method for steady state circumstances. A single thermodilution estimate of CO or SV has a COV or precision of 15–20%, while a triplicate injection technique has an error of 5% when the injections are made at different phases of the ventilatory cycle [19, 20]. It is against these levels of inherent error that any new method of CO or SV measurement should be judged [21]. However, the clinical circumstances of the critically ill patients with or without arrhythmia and in need for IABP often preclude extended hemodynamic steady state episodes, and exclude therefore a direct comparison between stroke volume by WKflow and thermodilution. Moreover during these rapid changing hemodynamics, mostly due to arrhythmia, there is a strong need for an actual continuous insight, which can only be given then by a beat-to-beat method. Therefore, we have chosen for a beat-to-beat comparison between WKflow method and the beat-to-beat left ventricular conductance volume method. The conductance method was per patient once calibrated by a triplicate phase spreading thermodilution technique [19, 20] during a relative stable episode. With this approach the effect of hemodynamic instability and arrhythmia is expected to be similar for SVlv and SVwf [14], which was uniformly observed see Fig. 6.

### Error analysis

Bland–Altman analysis for the difference between SVlv and non-calibrated SVwf resulted in percentage limits of agreement (LOA) of ± 23.2%. According to Critchley and Critchley [21] this percentage LOA is within the 30% limits of acceptability for clinical use. Because the conductance method and the WKflow method are physically independent of each other the error of the comparison C of two estimations A and B can be computed as Variance (C) = Variance (A) + Variance (B) [19, 20].

Kornet et al. [22], studied the accuracy of the conductance method to determine SV in comparison to thermodilution and estimated an error of around 10% for SVlv. The error we found for the difference between the SVwf and SVlv is on average 12% (Table 2, all). Thus we can conclude that SVwf method is responsible for an error of approximately √144–100 = 7%, which is comparable to the probable error for standard SV by thermodilution and of SVlv and may therefore replace the standard thermodilution method and conductance method. While the performance of SVwf is acceptable, after one thermodilution calibration per patient of WKflow the precision of the difference between SVlv and SVwf decreases to 7.8% (Table 2). Moreover, apart from quantitative estimation of SV, we observed an excellent agreement between SVlv and SVwf measures for directional SV changes, equal for calibrated and uncalibrated values, demonstrating that the SVwf can reliably track trends in SV as well as in CO.

### Use of aortic pressure from IAB catheter

In general pulse contour methods use the radial or femoral artery pressure as input for their calculation. This assumes that the radial or femoral pressure represents the pressure in the aorta and that changes in waveform morphology are related to corresponding changes in blood flow. However, profound differences between aortic and peripheral pressures have been reported especially during periods shortly after return from cardiopulmonary bypass [23, 24]. We previously showed [20] that this resulted in significant outliers in pulse contour CO values. Hatib and colleagues [25] showed that the radial and femoral arterial pressure may decouple from the aortic pressure. Using the WKflow method and solid state aortic pressure measured on tip of the IAB catheter, instead of more peripheral arterial pressure measured with a fluid filled manometer system solves this potential source of error.

A clean and high fidelity pressure signal from the tip-transducers is mandatory for precise detection of the start and end of systole. Both beat-to-beat SVlv and SVwf rely heavily on these beat-to-beat points in time. The start of systole by the WKflow method is defined as the time of the last local minimum in the arterial pressure before the start of the steep increase in arterial pressure. To detect the end of systole from arterial pressure several methods were proposed. The WKflow method which we previously used in the dicrotic notch detection algorithm [8, 9, 17, 18] and used in this evaluation detects the moment of aortic valve closure by identifying the first local minimum after peak in simulated aortic blood flow. Thus the WKflow software accurately detects begin and end of systole both during regular and irregular heartbeats. Therefore, we expect our estimates of both SVlv and SVwf to be reliable.

### Limitations

Because many heart failure patients frequently show arrhythmia, a new method should be tested also in this condition. Based on our analysis during periods of arrhythmia we conclude that if the IAB is properly timed to heart irregularities [9] then a reliable determination of beat-to-beat stroke volume is possible with this new WKflow method.

A potential limitation of the method is the patient selection. Similarly to the standard radial/femoral/finger arterial pulse contour methods, it is required that patients have a competent aortic valve and no aortic aneurysms which are both contraindications to IABP use. All patients studied had no signs of aortic valves leakage and aortic aneurysms. An aortic aneurysm may affect a patient’s arterial compliance. A competent aortic valve is needed because WKflow computes the forward flow into the aorta and ignores backward flow. For the analysis of conductance stroke volume an extra precondition is the absence of mitral valve regurgitation. We cannot exclude the possibility that some patients had small undetected valve leakages or that such leakages occurred during some periods of the observations. If so, this will lead to an overestimation of real stroke volume by WKflow during aortic regurgitation, an overestimation by the conductance volume method during mitral valve regurgitation. Furthermore, in our patients with LV aneurysm, marked LV dyssynchronic segmental volume changes were observed throughout the cardiac cycle, indicating diastolic and systolic dyskinetic and akinetic wall motions. The SV estimation by the conductance catheter is the sum of all segmental volume changes and the paradoxical segmental volume changes are consequently subtracted from total ventricular volume changes. On that way total ventricular SV estimation by including the dyssynchronous aneurysm segments will result in a valid SV estimation [26].

All our observations were carried out during cardiac surgery in patients with severe heart failure in whom a prophylactic use of IABP was advisable. Therefore, our IABP observations could only be done during episodes of minor surgical intervention.

## Conclusions

We found agreement between the results of beat-to-beat stroke volume values from the calibrated conductance method and non-calibrated WKflow method obtained during vena cava occlusion with and without IABP support. The two methods are interchangeable with respect to measuring absolute stroke volume as well as in monitoring changes in stroke volume. The precision of the new non-calibrated WKflow method is 11.6%. After one calibration per patient this precision improves to 7.8%. Incorporating the new WKflow algorithm into an IABP device allows monitoring of stroke volume or cardiac output on a beat-to-beat base allowing clinicians’ to quantify the impact of IABP therapy in real time. Subsequently it may provide an early confirmation of the effectiveness of the applied IABP settings and may indicate need for additional mechanical circulatory support therapy.
